# Sudden Unexplained Death in Childhood: A Neuropathology Review

**DOI:** 10.3389/fneur.2020.582051

**Published:** 2020-10-16

**Authors:** Declan McGuone, Laura G. Crandall, Orrin Devinsky

**Affiliations:** ^1^Department of Pathology, Yale School of Medicine, New Haven, CT, United States; ^2^Comprehensive Epilepsy Center, New York University School of Medicine, New York, NY, United States; ^3^SUDC Foundation, Herndon, VA, United States

**Keywords:** sudden death, SUDC, SIDS, neuropathology, hippocampus, sudden unexplained death in childhood

## Abstract

Sudden Unexplained Death in Childhood (SUDC) is the unexpected death of a child over age 12 months that remains unexplained after a thorough case investigation, including review of the child's medical history, circumstances of death, a complete autopsy and ancillary testing (1). First defined in 2005, SUDC cases are more often male, with death occurring during a sleep period, being found prone, peak winter incidence, associated with febrile seizure history in ~28% of cases and mild pathologic changes insufficient to explain the death (1, 2). There has been little progress in understanding the causes of SUDC and no progress in prevention. Despite reductions in sudden unexpected infant death (SUID) and other causes of mortality in childhood, the rate of SUDC has increased during the past two decades (3–5). In Ireland, SUID deaths were cut in half from 1994 to 2008 while SUDC deaths more than doubled (4). Surveillance issues, including lack of standardized certification practices, affect our understanding of the true magnitude of unexplained child deaths. Mechanisms underlying SUDC, like SUID, remain largely speculative. Limited and inconsistent evidence implicates abnormalities in brainstem autonomic and serotonergic nuclei, critical for arousal, cardiorespiratory control, and reflex responses to life-threatening hypoxia or hypercarbia in sleep (6). Abnormalities in medullary serotonergic neurons and receptors, as well as cardiorespiratory brainstem nuclei occur in some SUID cases, but have never been studied in SUDC. Retrospective, small SUDC studies with non-standardized methodologies most often demonstrate minor hippocampal abnormalities, as well as focal cortical dysplasia and dysgenesis of the brainstem and cerebellum. The significance of these findings to SUDC pathogenesis remains unclear with some investigators and forensic pathologists labeling these findings as normal variants, or potential causes of SUDC. The development of preventive strategies will require a greater understanding of underlying mechanisms.

## Introduction

Sudden unexplained death in childhood, (SUDC) is the sudden and unexpected death of a child 12 months or older that remains unexplained after a thorough case investigation, including review of the child's medical history, circumstances of death, a complete autopsy and ancillary testing (1). It is the 5th leading category of death in children aged 1–4 years, and resulted in 243 deaths in 2017 (7). The rate of SUDC is increasing, even as mortality from sudden unexpected infant death (SUID) continues to fall (3–5). In Ireland, SUDC deaths more than doubled from 1994 to 2008, a period when SUID rates declined by 50% (4).

The paucity of SUDC literature precludes a detailed protocol-driven systematic review of the topic: published reports consist predominantly of limited autopsy-based case series or epidemiologic observations from a limited number of registries and cohorts. A PubMed search (08/10/2020) for sudden infant death syndrome or SUID revealed 15,355 articles but only 31 results for SUDC. Further, surveillance issues, including lack of standardized certification practices, and non-specific coding (R96 or R99) limit our ability to accurately assess SUDC incidence. These cases challenge the under-resourced and non-standardized U.S. medicolegal death investigation system, leaving a devastating impact on affected families (8, 9). To date, there has been little progress in understanding the causes or preventing SUDC.

## Clinical and Genetic Features of SUDC

SUDC is a diagnosis of exclusion that refers to a heterogeneous group of underling conditions. Phenotypic overlay with (SUID) and sudden unexpected death in epilepsy (SUDEP), suggests a biologic continuum between these sudden death syndromes (Table 1). A risk profile for SUDC has emerged over the past decade; most reported cases affect white non-Hispanic boys who were the product of a full-term pregnancy, with a median age of 1.6 years, (range 1–3 years) (1, 8). Population-based data of all-cause mortality affecting children aged 1–4, identifies ill-defined or unknown causes of death (R99), twice as frequently in African American children, underscoring that some SUDC cohorts have suffered selection bias in case ascertainment (7). Deaths are usually unwitnessed, occur during apparent sleep, and most children are discovered in a prone position, often face down (11). A history of febrile seizures (FS) is reported in up to one third of SUDC cases, (vs. ~2–4% of controls); the median age at death tends to be greater in children with a FS history compared to those without, (24 vs. 19 months) (12). A high prevalence of FS history across SUDC cohorts generally comports with a spectrum of neuropathologic hippocampal observations of unclear biologic significance (Table 2). Not all SUDC cases with FS history are associated with hippocampal changes suggesting other mechanisms are likely relevant in some instances (12, 16). FS history might represent an independent marker for SUDC, with the caveat that not all FS are clinically obvious, and FS are probably underreported (2, 17). The estimated FS prevalence of ~1–2% in the general pediatric population, and a low overall SUDC annual incidence, suggests additional factors modulate risk of SUDC in susceptible children, although data are limited regarding specific genetic variants influencing risk. As non-motor seizures may be associated with life-threatening apnea in early childhood the possibility that some SUDC cases represent SUDEP in children with undiagnosed epilepsy cannot be excluded (10). Further, a witnessed FS history is more frequent among explained pediatric deaths than children in the general population, although terminal seizures triggered by an exogenous stressor such as infection might still be relevant in these cases (2). A history of minor illness or fever in the 48 h prior to death, prior infection, minor head trauma, and peak winter incidence have also been associated with SUDC (1, 3, 18). By definition, autopsy examination and ancillary studies are negative or reveal only minor pathologic changes insufficient to explain death. Thus, detection of confirmed pathogenic variants by whole exome sequencing, such as cardiac channelopathy-susceptibility genes encoding sodium, potassium, or intracellular calcium channels, represent autopsy cases that become explained by genetic findings and are thereby excluded from a SUDC category of death (8). Pathogenic cardiac variants have been reported in up to 25% of sudden deaths lacking gross anatomic findings at autopsy (19–21). Although the genetic factors influencing SUDC vulnerability remain largely unknown, similarities with SUID and SUDEP, suggest seizure or cardiac related mechanisms are relevant in many cases. Moreover, exome sequencing of SUDEP cases has identified an excess of variants in genes that regulate ion channels in cardiac and brain tissue (22, 23). Perturbations of normal brain development resulting from *de novo* somatic mutations during embryonic or early post-natal development are increasingly recognized in multiple neurodevelopmental disorders including migration defects, epileptic encephalopathies, and other neuropsychiatric conditions, although a causal role in SUDC remains to be demonstrated (24, 25).

**Table 1:** Phenotypic features of SUID, SUDC, and SUDEP (10).

	**SUID**	**SUDC**	**SUDEP**
Male Sex	+	+	+
Age	<1yr	1–5 years	Any age
Death during apparent sleep	++	++	++
Prone position	++	++	++
Seizure history	+/–	+(FS)	++
Preterm birth/low birth weight	+	+/–	Unknown
Bedsharing/unsafe sleep environment	+	Unknown	Unknown
Illness/fever <48 h before death	+	+	–
Smoke exposure	+	Unknown	Unknown
Autopsy	WNL	WNL	WNL
Hippocampal microscopic changes	+	+	+
Serotonergic abnormalities	+	Unknown	+

*SUID, Sudden unexpected infant death; SUDC, Sudden unexplained death in childhood; SUDEP, Sudden unexpected death in epilepsy; WNL, (within normal limits; minor findings insufficient to cause death are common); FS, febrile seizures; Associations are positive (+), strongly positive (++), or negative (–)*.

**Table 2:** Relative frequency of hippocampal findings and associated changes in SUDC in published series.

**Year**	**References**	**Dataset**	**Age (months) Median/range**	**Hippocampal abnormalities**	**Ethnicity**	**Gender**	**FS history**	**Asymmetry/** **malrotation**	**Microscopic DG alterations**	**FDGB**	**Irreg** **DG**	**Ectopic GCs**	**GC** **loss**	**Subicular abnorm**	**Hipp** **Nn loss/** **gliosis**	**Other NP** **findings**
2007	Kinney et al. (3)	SDSRP	18/13–36	5/23	4C; 1C/A	4M;1F	2/5	5/5	4/5	–	–	–	–	3/5	0/5	SVN, Co. NeoCx, MD
2009	Kinney et al. (13)	SDSRP	20.4/12–70.8	16/26	13C; 2A; 1 other	8M;8F	7/16	5/26	10/26	–	–	–	–	4/26	2/26	IOD (3/16); FLC (2/16); WM and PAG heterotopia, (2/16)
2016	Kinney et al. (14)*	SUDP at BCH; SDSRP	23.7–44.9	17/17	17C	10M;7F	11/17	–	17/17	16/17	10/17	17/17	16/17	7/17	8/17 (HG)	FCD (9/16); Hamartia (4/17); heterotopia (8/17); IOD (3/16); OH (7/16); Arcuate MD (5/16); cerebellar MD (3/16); FP (3/16)
2016	Hefti et al. (16)	SDSRP; BCH	12–79.1	42/79	64C	43M;34F	24/77	17/42	63/79	38/79	48/79	63/79	55/79	–	43/79 (HG)	WM heterotoia (5/77); IOD (1/49); encephalitis (1/77); HIC (22/77)
2020	McGuone et al. (15)	SUDCRRC	33.3/12–142	19/20	16C; 3H/W; 1EA/H/W	8M;12F	12/20	0/20	19/20	3/20	6/20	3/20	6/20	0/20	0/20	Incidental minor findings (2/20)

FS, personal febrile seizure history; DG, dentate gyrus; FDGB, focal dentate gyrus bilamination; GC, granule cell; Nn, neuronal; NP, neuropathologic; SDSRP, San Diego SUDC Research Project; SUDP, Sudden Unexplained Death in Pediatrics Program; BCH, Boston Children's Hospital; SUDCRRC, SUDC Registry & Research Collaborative; C, Caucasian; A, Asian; AA, African American; H, Hispanic; EA, East Asian; M, male; F, female; *age 1 to <6 yrs; HG, hilar gliosis; SVN, subventricular neuroblasts; Co. NeoCx. columnar neocortex; MD, microdysgenesis; IOD, inferior olivary dysplasia; FLC, fetal like cortex; WM, white matter; PAG, periaqueductal gray; FCD, focal cortical dysplasia; OH, olivary heterotopia; FP, fused pyramids; HIC, hypoxic ischemic change.

## Neuropathologic Findings in SUDC

SUDC likely represents a phenotypic endpoint for a heterogeneous group of underlying disorders, the mechanisms of which remain poorly defined. The proportional contribution of central nervous system disorders to this shared phenotype is unknown. A complete autopsy is the gold standard for understanding causes and consequences of lethal disease, and a detailed examination of the brain is necessary to identify potentially unexpected neurologic causes of death. This is also critical to power research to inform future preventative interventions. However, in the United States sudden unexpected pediatric deaths are investigated by a non-uniform medico-legal investigation system consisting of over 2,000 autonomous jurisdictions run by a mixture of physician medical examiners and lay coroners (26). At a diagnostic level an absence of procedural guidelines for pediatric death investigation beyond infancy, combined with uneven access to pediatric and neuropathology expertise has resulted in large variation of autopsy standards, with neuropathologic examinations that are frequently insufficient. Finally, although essential for public health surveillance, the medical death investigation system is under-resourced, short-staffed, and chronically under-funded, problems which have deepened during the opioid epidemic, and COVID-19 novel coronavirus disease pandemic. Together, these issues have conspired to severely limit progress in understanding SUDC pathogenesis.

One audit of SUDC autopsy practice found improved reporting when autopsies were performed by pediatric pathologists compared to non-specialists (27). Protocols for the investigation of unexpected infant death and recently published guidelines for SUDC are available; however, implementation remains problematic due to the persistent and systematic challenges described above (8, 28–31).

Neuropathologic findings in SUDC are particularly relevant when a seizure is postulated as the immediate mechanism of death. There are no specific autopsy findings to confirm an acute seizure, and, if a death is unwitnessed, as occurs in more than 90% of cases, the diagnosis becomes speculative without strong supporting circumstantial evidence (32). However, stigmata of convulsive seizures may be absent. General autopsy findings such as tongue biting and urinary incontinence are occasionally seen in older children and adults; however, these only occur in a minority of cases and lack specificity (2, 33–35). Further, young children can have non-convulsive seizures that cause respiratory arrest and near-death events (36, 37). Finally, even in adults with epilepsy, sudden death can occur during video electroencephalogram (EEG) monitoring without clinical or electrographic evidence of a seizure, and autopsies reveal no alternative causes.

Neuropathological research has emphasized a biologic continuum between SUDC and SUID, focusing on two inter-connected brain regions: the hippocampal formation, a limbic system hub connecting to brainstem in the central autonomic network, and brainstem cardiorespiratory centers (38–40). Brainstem serotonergic and autonomic nuclei are critical in controlling arousal as well as cardiorespiratory centers that respond to life-threatening hypoxia or hypercarbia during sleep. Although extensively studied in SUID, in older children research has instead focused mainly on the hippocampal formation (6, 41, 42). In SUDC, neuropathological studies have focused on hippocampal abnormalities, yet no study has examined the role of medullary serotonergic brainstem neurotransmission (14, 43).

**Figure 1:**
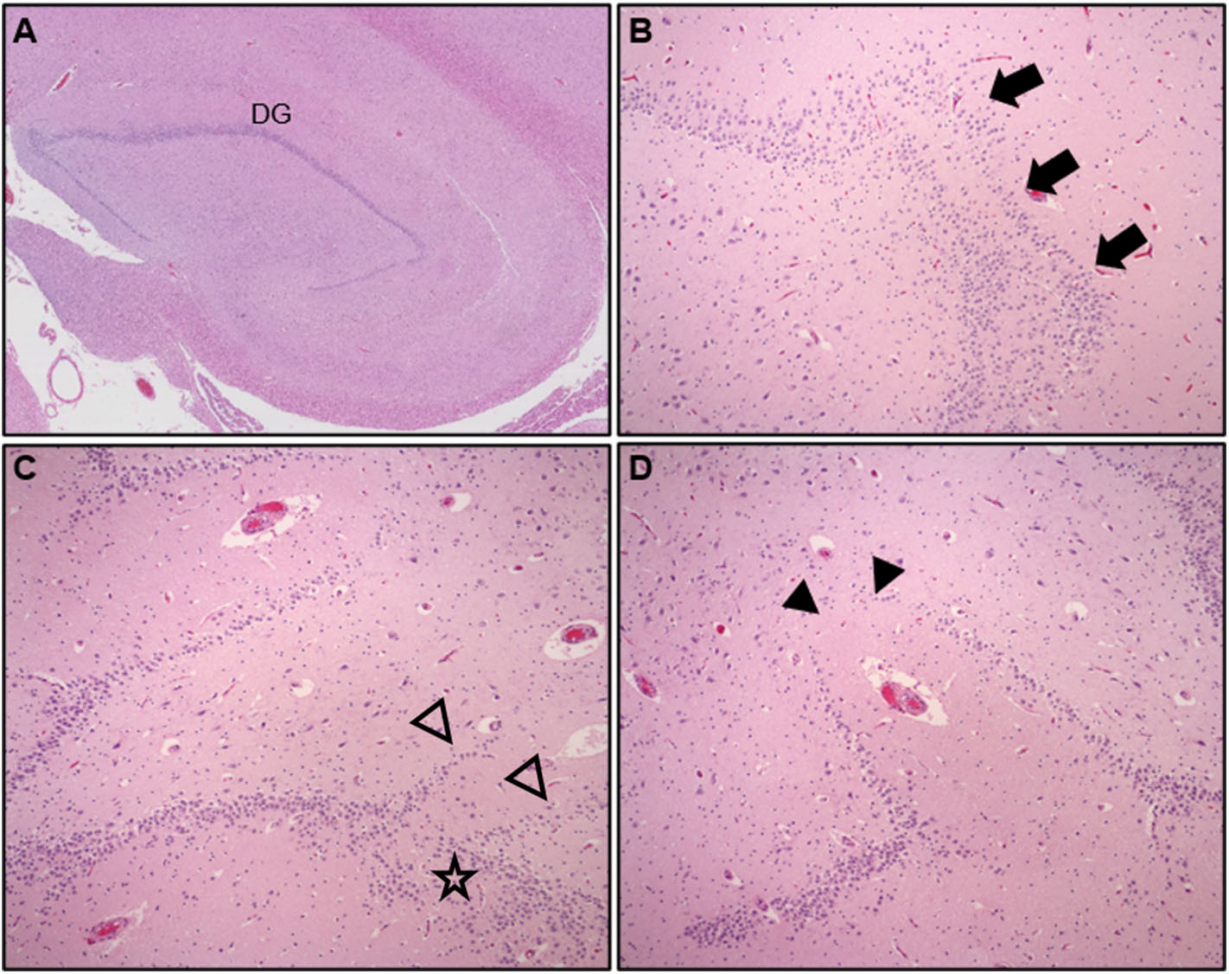
Hematoxylin & Eosin (H&E) stained hippocampal tissue sections at lateral geniculate nucleus level showing **(A)** compact arrangement of the normal dentate gyrus (DG), low power, **(B)** granule cell dispersion (GCD), with focal dentate gyrus bilamination, arrows (mag = 200X), **(C,D)** adjacent hippocampal sections from a child with explained cause of death showing complex DG architecture, abnormal linear bands of granule cells (open arrowheads), GCD, (star), and focal DG granule cell loss, (arrowheads), (mag = 200X).

Although epidemiologic data support a link between neuropathologic changes, FS history, and SUDC, the nature of this association is poorly understood. Early exploratory analyses of the San Diego SUDC Research Project, (SDSRP), a multicenter initiative created to characterize the main pathologic features and risk profile of SUDC, were key to elucidating the initial relationships between external and microscopic abnormalities of the hippocampus, sudden death during apparent sleep, and FS history (1, 3, 13). Subsequent analyses have expanded on this original hippocampal phenotype to identify the key elements of Hippocampal Malformation Associated with Sudden Death, (HMSASD) (16). The defining features of HMASD include external malrotation or asymmetry of the hippocampus, and a cluster of developmental lesions centered on the dentate gyrus (DG) (Table 2). Additional analyses have emphasized alterations of the granule cell layer (GCL) including granule cell dispersion (GCD) and focal dentate gyrus bilamination (FDGB) as key findings (Figure 1) (14, 40). Similar GCL alterations occur in hippocampal sclerosis in temporal lobe epilepsy, where FDGB is associated with more severe disease (44). Whether these changes are necessary or sufficient to cause seizures in SUDC remains unproven and controversial (16, 45, 46). Unlike temporal lobe epilepsy, hippocampal sclerosis is rare or never occurs in SUDC while acquired hippocampal injury (e.g., neuronal loss or gliosis) is uncommon in SUDC (13, 15, 16).

The strong association between hippocampal alterations and FS history has prompted speculation that the mechanism of SUDC could be a terminal seizure-like event, reminiscent of sudden unexpected death in epilepsy (SUDEP) (2, 12). A key unanswered question, however, is the biologic relevance of GCL alterations as it remains unclear whether these changes are a cause or a consequence of seizures. Moreover, the extent to which GCL alterations overlap with normal anatomic variation requires further clarification. Evidence in support of a developmental basis of hippocampal changes is inferred primarily from imaging and autopsy data. Rare case reports of bilateral GCD in infancy show an association with cortical polymicrogyria in some cases, although EEG data and seizure history were lacking (47). Many autopsy reports are limited by insufficient correlative clinical data and subclinical seizures usually cannot be excluded, raising doubts as to the strength of the evidence in support of a developmental hypothesis. Additionally, inconsistent histologic classification schemes, absence of agreed upon definitions, and non-uniform sampling protocols make subjective interpretations of GCL alterations problematic without additional and detailed morphometric studies (15, 48). Some experimental data suggest seizure induced neurogenesis and altered neuronal migration as playing a causal role in GCL alterations (49–51). However, other experimental data suggest GCL alterations as causal in seizure genesis, so the question of cause vs. effect remains unresolved (52, 53).

A retrospective analysis of a large SUID cohort in an urban medical examiner setting, found FDGB in ~40% of infants, and ~8% of explained deaths (43). A similar distribution of GCL alterations has consistently been reported in explained “control” groups of SUDC cohorts (15, 16). The apparent high prevalence of GCL alterations in pediatric deaths with explained causes raises questions about the etiologic specificity, and therefore biologic relevance, of the role of GCL alterations in the SUDC brain. Observational and experimental protocols designed to evaluate GCL alterations are necessarily biased toward hippocampal sampling which might account for a trend toward increased sensitivity to over-interpret normal anatomic variants in some cases as the spectrum of normal variant anatomy during hippocampal development is poorly defined and prevalence data for the general pediatric population are lacking. A recent morphometric analysis of archived autopsy pediatric material found no correlation between GCD and seizure history (54). Thus, the significance of GCL alterations remains uncertain.

Autopsy data are an endpoint and have limited capacity to inform our understanding of the natural history of a disease process. However, the frequent association of GCL alterations and FS history in SUDC has prompted some researchers to suggest that GCL is a marker of seizure vulnerability in early life, potentially through age-dependent mechanisms involving altered limbic-brainstem connections (38). Multiple hippocampal structural abnormalities were reported in the most recent SDSRP cohort report (16). Half of SUDC cases showed HMASD with or without a FS history and the most frequent histologic findings in this group were GCD, FDGB, irregularity of the DG, and ectopic granule cells. The investigators suggested that a malformed hippocampus could predispose to seizure development during sleep when seizure risk is greatest, however, this analysis also revealed comparable SUDC rates in cases that lacked hippocampal changes, raising doubts whether this finding is signal or noise. Notably, almost half of the cohort did not have hippocampal alterations and 27% did not have febrile seizures. However, researchers have since raised concerns about the reliability of the association in the SDSRP study, as hippocampal tissue was unavailable for analysis in approximately half the cases (46). Other methodologic issues included an incomplete dataset populated by self-referred cases that lacked standardized death investigation, with frequent limited brain examination and sampling (46, 55).

Standardized, unrestricted whole brain examination is essential to elucidate the structural abnormalities, if any, in the SUDC brain. Thus, the SUDC Registry and Research Collaborative (SUDCRRC) at NYU Langone Health undertook a 5-year prospective analysis of 20 SUDC cases accrued through a registry where systematic sampling of whole brains was performed with blinded independent reviews conducted by neuropathologists (15). The observations were supplemented by 3T-MRI imaging, and whole exome sequencing to identify pathogenic variants relevant to death. Whole brain analysis revealed hippocampal alterations of the DG as the most frequent microscopic alteration across all examined brain regions, although these were not specific to SUDC as three cases with these findings died from pathogenic genetic cardiac variants. The most frequent DG alterations included alternating thickness, irregular configuration, focal GCL loss, and ectopic neuronal clusters. Unlike prior reports, GCD or FDGB, were not prominent findings, raising questions about the importance of this finding as a morphologic marker of SUDC in this cohort. It was not immediately apparent why GCL alterations were not prominent in this series as 30 individual hippocampal observations (15 on either side) were scored based on the defining features of HMASD (14). As morphometric analysis was not conducted the possibility of heightened sensitivity and observer bias to specific lesions, particularly in more subtle cases, cannot not be entirely excluded. Alternatively, and perhaps more likely, is that prior studies suffered observer bias as there was greater tendency to consensus-based decision making without blinding.

A history of FS was present in ~60% of these SUDC cases, substantially more than previously reported cohorts and likely reflecting referral bias from forensic institutions with limited access to neuropathology expertise. The registry provided *ex vivo* MRI imaging and brain examination by a board-certified neuropathologist. A history of subclinical seizures could not be excluded in one patient with FDGB who had no FS history. Importantly, this study showed no consistent distribution of microscopic findings outside the hippocampus, such as cerebellar cortical dysplasia and anomalous inferior olivary nuclei, findings which were occasionally seen in other cohorts. Although a well-conducted prospective cohort study, this study still suffered limitations. The small sample size due to the rarity of SUDC, limited access to true normal controls such as pediatric trauma, or children without medical comorbidities, and a hippocampal sampling bias, necessarily require that conclusions about the relative contribution of hippocampal abnormalities in SUDC should still remain tentative.

Neuropathologic research in SUDC has focused primarily on the hippocampus, with speculative mechanisms of a limbic-brainstem network disorder contributing to epileptogenesis and brainstem dysfunction in susceptible children (48, 56). These hypotheses remain untested and the brainstem is conspicuously understudied in SUDC. Multiple neurotransmitter defects of brainstem respiratory and autonomic pathways were identified in SUID brains, with abnormalities of the medullary 5-Hydroxytryptamine (serotonin) (5-HT) system implicated as a major network vulnerability for sudden death in infancy (6). Notably, the same group that has championed the role of hippocampal abnormalities in SUDC and SUID had previously highlighted the role of medullary serotonin and other brainstem abnormalities, including structural abnormalities in the inferior olive, reactive gliosis in the medulla, interleukin 6 and nicotinic and muscarinic receptor abnormalities in the medulla (57–59).

Medullary 5-HT neurons are central respiratory chemosensors and contribute to arousal and key autonomic functions (41, 60). Further, altered serotonergic transmission is altered in animal models of hippocampal dysfunction and epilepsy, and serotonin efferents from brainstem raphe neurons to the DG regulate GCL neurogenesis and cell migration in early hippocampal development (43, 61). It remains unclear whether hippocampal structural changes in SUDC can result from disturbed neurotransmission due to an intrinsic brainstem serotonergic defect arising during early development. Further research is necessary to clarify the significance of limbic-brainstem connections in SUDC, and whether GCL alterations could represent a potential marker of underlying 5-HT brainstem defects. The challenge is that studies typically focus on one or two anatomical structures or functional assays, and the brain has hundreds of networked structures and scores of neurotransmitters, neuromodulators and receptors. Viewing a tiny fraction of a complex picture may lead to spurious conclusions perhaps most dangerous, they may close the door to more relevant pathogenic structures or functional systems.

## Potential Neurologic Mechanisms of SUDC

The functional basis of SUDC remains poorly defined partly because witnessed deaths are extremely rare, but also because relevant animal models are lacking. SUDC is an endpoint for diverse disorders, some of which may be seizure driven and display phenotypic overlay with SUDEP. To date, the only one witnessed SUDC case report was a 20-month-old toddler undergoing epilepsy monitoring in whom febrile status epilepticus was followed by bradycardia (2). One proposed mechanism of SUDC associated with FS includes thermal sensitivity of the developing brain central homeostatic network (40). The potential for seizure-like events precipitated by exogenous stressors remains speculative and shares similarities with the triple risk model of SUID (5). Although the range of potential stressors is unknown, epidemiologic data have identified that SUDC occurs more frequently in winter, and ~75% have a history of a recent minor illness within 2 weeks before death (1, 11). Moreover, minor inflammatory infiltrates are common in the lungs and other organs at autopsy, suggesting that post-infectious immunologically mediated processes could also be relevant, although these findings are not specific and occur commonly in other children with well-explained causes of death (1). A history of minor blunt head injury identified in one quarter of SUDC cases from the initial SDSRP cohort, suggested a potential role for post-concussive mechanisms, although trauma has not been reported as a correlative risk for SUDC in subsequent analyses (1). Retrospective questions of parents who have lost a young child may also be biased by a desire to “find the answer,” potentially biasing recall of minor head injuries as more serious. In addition, shared circumstantial features of early childhood deaths, including a tendency for death to occur in a prone position during a period of apparent sleep suggests pathophysiologic mechanisms related to sleep and development might be important, at least in some instances.

SUDC and pediatric SUDEP share several features (14, 40, 62). SUDEP is a diagnosis of exclusion that refers to the sudden, unexpected death of a person with epilepsy in whom an autopsy does not reveal a structural or toxicologic reason for death (63). Seizure-induced autonomic or respiratory disturbances are implicated in most SUDEP deaths (10, 48). The age of epilepsy onset influences SUDEP risk, which increases with earlier onset epilepsy, implying that developmental mechanisms might influence vulnerability (62, 64). Hippocampal anomalies of the DG, analogous to SUDC, occur in some SUDEP brains, but are not common in SUDEP and many epilepsy patients who die from other causes have hippocampal abnormalities (32). These changes also occur in experimental animal models where hippocampal stimulation evokes cardiorespiratory effects (48, 65–67). In some cases, a distinction between SUDC and SUDEP may be semantic as some SUDC cases had epilepsy syndromes retrospectively diagnosed based on genetic and other data, but epilepsy was not recognized before death.

The mechanisms of death in SUDEP are poorly understood because few observed cases have occurred during epilepsy monitoring. Most SUDEP cases occur during apparent nocturnal sleep and multifactorial mechanisms likely include seizure-induced autonomic disturbance, impaired arousal, apnea, cardiorespiratory depression, and brainstem dysfunction (10). The role of these heterogeneous mechanisms in SUDC pathogenesis remains poorly defined. The Mortality in Epilepsy Monitoring Units Study, (MORTEMUS), identified 11 SUDEP patients who died after terminal generalized tonic-clonic seizures during epilepsy monitoring (68). Cardiorespiratory data were available for 9 patients who displayed a consistent pattern of seizure associated cardiorespiratory collapse that included postictal tachypnea and cardiac dysfunction followed by central apnea, severe bradycardia, and asystole. Imaging and volumetric assessments of SUDEP brains have demonstrated volume changes affecting brain regions responsible for modulating cardiorespiratory control, including atrophy in areas that protect against cardiorespiratory collapse, and increased volume in areas that predispose to apnea and/or hypotension (69, 70). Comparable volumetric analyses in SUDC cohorts are lacking. As with SUID, abnormalities of the serotonergic system and other neurotransmitter systems are implicated in SUDEP; the 5-HT type 2c receptor knockout mouse is an animal model of SUDEP with spontaneous seizures leading to respiratory arrest and death within seconds of onset (10, 71). Medullary serotonergic neurons function as chemosensors responsible for stimulating breathing and arousal in response to hypercapnia and may contribute to ictal apnea and respiratory arrest if inhibited by seizures (72, 73).

Although the role of serotonergic network dysfunction in SUDC pathogenesis remains unknown, the requirement for hippocampal transmission from brainstem serotonergic neurons for early DG neurogenesis suggests hippocampal alterations may be a morphologic marker of altered brainstem serotonergic function (43, 61). Given these converging strands of evidence, hippocampal abnormalities have emerged as a potential risk factor for seizure-like mechanisms responsible for SUDC, potentially mediated through apneic seizures and cardiorespiratory dysregulation (40). Some researchers suggest these changes might be considered as “pre-epilepsy” because of a presumptive increased risk for seizure progression, a lesion termed “epilepsy *in situ*” (65). However, there is limited evidence to directly support the role of hippocampal dysfunction in SUDC pathogenesis. The structural changes in the hippocampus may be secondary to interictal or ictal discharges (44).

## Epidemiologic Features of SUDC

Understanding risk factors and the underlying biologic mechanisms of SUDC is essential for informing effective public health surveillance and developing strategies to mitigate risk in susceptible children. Successful educational and public health campaigns have substantially reduced the burden of SUID (11, 74). In contrast, SUDC has received relatively little attention, partly because it is such a rare condition The Sudden Death in the Young registry, under the auspices of the Centers for Disease Control and Prevention and the National Institutes for Health is responsible for tracking unexplained infant as well as childhood deaths, although just 13 states or jurisdictions actively participate in this program (75, 76). Further, the low incidence of SUDC creates additional challenges for healthcare providers who often rely on an interdisciplinary approach to recently bereaved families, and interface with the medico-legal system (8, 11).

Before 2005, few studies considered unexplained post-infancy death other than brief descriptions in occasional medico-legal series primarily concerned with distinguishing natural from unnatural death (77–80). Brain findings were generally not discussed unless gross pathologic abnormalities were seen; i.e., hippocampal histology was not reported. However, many of these cases would likely be re-classified in the modern era through contemporaneous genomic analyses. Molecular testing has yielded high rates of pathogenic cardiac variants in up to 25% of cases of published sudden death cohorts, and ~12% of explained sudden pediatric deaths which possibly explain cause and mechanism of death in some cases, underscoring the necessity of comprehensive ancillary testing to identify causes (15, 20, 21). Moreover, non-neurologic or cardiac mechanisms may also account for a subset of SUDC deaths.

A large population study of ~10,000 children enrolled in a SUID study identified 5 unexplained deaths in children 1–5 years of age (81). Two deaths were associated with apnea and convulsions. Another retrospective analysis of childhood mortality by pathologists in England found 5 unexplained deaths among 1,012 sudden pediatric deaths, although brain findings were not reported for unexplained cases (82). Other studies have identified similar trends, and countries that routinely track SUDC mortality report a gradual increase in incidence over time (4, 83–85).

## Conclusions

SUDC is a major cause of unexpected mortality in toddlers that devastates families. Clinicopathologic correlation remains elusive as SUDC lacks a distinct pathoanatomic signature—in the brain or elsewhere. Most SUDC fatalities are unwitnessed and little is known of the immediate pathophysiologic disturbances preceding death, although seizure-like mechanisms analogous to SUDEP may explain some cases with a FS history (12). Accrual of SUDC cases through SUDC registries has enabled researchers to construct a predicted profile of a typical SUDC child, although this does not capture the heterogeneity of underlying disorders that are likely responsible for sudden death in children. Retrospective studies with non-standardized methodologies have demonstrated a high rate of hippocampal abnormalities associated with FS history, and SUDC. These studies have been limited by sample size and lack of appropriate controls, which limit conclusions that can be drawn. Future detailed studies are necessary to elucidate the mechanism of hippocampal alterations, and explore connections with brainstem dysfunction. A developmental continuum has been proposed as a framework for SUID and SUDC, with the expectation that future advances in imaging and genomics will help resolve convergent mechanisms and pathways of relevance to both groups (39). Ultimately additional and adequate support for a resource-limited medicolegal death investigation system is necessary to allow the extensive ancillary testing required to fully investigate and identify and exclude underlying pathologies in putative SUDC deaths. Greater interdisciplinary participation in research efforts is also crucial to elucidate underlying mechanisms, identify children are at increased for sudden death, and to institute appropriate screening and preventative strategies.

## Author Contributions

All authors were involved in the conception, literature review, writing, and editing.

## Conflict of Interest

LC holds a volunteer board position at the SUDC Foundation. The remaining authors declare that the research was conducted in the absence of any commercial or financial relationships that could be construed as a potential conflict of interest.
